# X-Linked Nephrogenic Diabetes Insipidus Associated with the AVPR2 c.964C>T (p.Pro322Ser) Variant: A Family Case Series

**DOI:** 10.3390/jcm15145514

**Published:** 2026-07-14

**Authors:** Kalliopi Vardaki, Ioannis Petrakis, Eleni Drosataki, Christos Pleros, Ariadni Androvitsanea, Dimitra Lygerou, Kleio Dermitzaki, Antonakis Andreas, Konstantina Kydonaki, Kostas Stylianou

**Affiliations:** 1 Department of Nephrology, Heraklion University Hospital, 71500 Iraklio, Crete, Greece; petrakgia@gmail.com (I.P.); elenidro2@hotmail.com (E.D.); xpleros@gmail.com (C.P.); ariaandrovitsanea@gmail.com (A.A.); dimitra.ligerou@gmail.com (D.L.); ekderm@gmail.com (K.D.); antonakisandreas@yahoo.gr (A.A.); k.ntina@hotmail.com (K.K.); kstylianu@uoc.gr (K.S.); 2 Department of Pediatrics, Heraklion University Hospital, 71500 Iraklio, Crete, Greece

**Keywords:** nephrogenic diabetes insipidus, AVPR2, p.Pro322Ser, X-linked inheritance, genotype–phenotype correlation, phenotypic variability

## Abstract

**Background:** Nephrogenic diabetes insipidus (NDI) is a rare disorder characterized by renal resistance to arginine vasopressin, most commonly caused by pathogenic variants in the AVPR2 gene. While X-linked NDI classically affects males, heterozygous females may exhibit variable clinical expression. Certain AVPR2 variants are associated with partial NDI and milder phenotypes. **Methods:** We conducted a retrospective family study of a multigenerational Greek pedigree with suspected hereditary NDI. Clinical, biochemical, and pedigree data were collected through chart review and family interviews. Genetic analysis was performed using whole-exome sequencing, and variant interpretation followed ACMG/AMP guidelines. **Results:** Fourteen individuals across four generations were evaluated. Molecular analysis identified a familial AVPR2 (NM_000054.7):c.964C>T (p.Pro322Ser) missense variant in three males and three females, with obligate carrier status inferred in two deceased females, segregating in an X-linked pattern. Hemizygous males exhibited a broad phenotypic spectrum, ranging from partial NDI with later onset to severe early-onset disease with urinary tract complications. Heterozygous females showed variable expression, from asymptomatic carriers to mildly symptomatic individuals. The variant co-segregated with disease and, based on ACMG criteria, it was classified as pathogenic. **Conclusions:** In our family, the AVPR2 c.964C>T (p.Pro322Ser) variant was associated with a remarkably broad clinical spectrum, ranging from asymptomatic heterozygous females to severe early-onset disease with urinary tract complications in affected males. These observations emphasize the need for early molecular diagnosis, systematic evaluation of female carriers, and long-term surveillance to prevent disease-related complications and optimize genetic counselling.

## 1. Introduction

Nephrogenic diabetes insipidus (NDI) is characterized by renal resistance to antidiuretic hormone (ADH; also named arginine vasopressin or AVP), leading to an inability to concentrate urine despite normal or elevated circulating ADH levels. Primary forms of NDI result from alterations in the genes that encode the key proteins AVPR2 and AQP2, whereas secondary forms have been associated with biochemical abnormalities such as hypokalemia and hypercalciuria [1,2], obstructive uropathy [3,4] or the use of certain medications, particularly lithium [5]. Most patients with congenital NDI present with failure to thrive and vomiting during the first year of life, whereas those with acquired NDI typically present later in life with polyuria and/or polydipsia. Laboratory investigations reveal the typical picture of hypernatremic dehydration with inappropriately dilute urine and dilatation of the urinary tract is a recurrently noted complication of NDI, especially for the patients with poor voiding [6].

Approximately 90% of hereditary NDI is caused by alterations in the AVPR2 gene, which is located on chromosome region Xq28, while most of the remaining cases are due to AQP2 variants, which are located on chromosome 1 [7,8]. Recent studies and international consensus statements continue to emphasize the predominance of AVPR2-related disease and the importance of genotype–phenotype correlations in guiding diagnosis and management of hereditary NDI [9,10]. To date, more than 300 different AVPR2 variants have been described [11,12]. The AVPR2 gene encodes the vasopressin V2 receptor, which is primarily expressed in collecting duct principal cells of the kidney. Binding of vasopressin to this receptor activates cAMP-dependent signaling and promotes aquaporin-2 trafficking to the apical membrane, thereby increasing water reabsorption. Loss-of-function variants in AVPR2 impair this process and cause X-linked NDI.

Although X-linked NDI caused by AVPR2 variants classically affects males, heterozygous females may be asymptomatic or exhibit variable degrees of polyuria and polydipsia, likely due to skewed X-chromosome inactivation. Recent reports continue to document symptomatic female carriers and further support the contribution of epigenetic mechanisms to phenotypic variability in X-linked NDI [13]. In addition, certain AVPR2 changes are associated with partial NDI, when kidney tubules show partial resistance to AVP, resulting in a milder or later-onset clinical presentation [14]. The p.Pro322Ser variant has been previously linked to partial NDI phenotype with normal urinary osmolality in affected individuals [15].

Here, we describe a Greek family with X-linked NDI associated with the AVPR2 NM_000054.7:c.964C>T (p.Pro322Ser) variant, highlighting the marked intrafamilial phenotypic variability observed across generations.

## 2. Materials and Methods

This was a retrospective family study based on clinical records and genetic testing of available family members. Fourteen individuals from four generations were included in the study. Clinical information was available for all family members, while molecular testing was performed in ten living individuals. Two additional deceased females were considered obligate heterozygous carriers based on pedigree analysis. Demographic, clinical and pedigree data were obtained through chart review and family interview. Collected variables included year of birth, age at presentation, clinical presentation, laboratory findings and any evidence of urinary tract complications. Genetic analysis was performed in the Nephrology Lab of the University of Crete, Greece. The study has been approved by the ethics committee of the University Hospital of Heraklion with a protocol number 2529/17-6-2015 (Approval date 17 June 2015) and performed in accordance with the ethical standards as laid down in the 1964 Declaration of Helsinki and its later amendments or comparable ethical standards. Written informed consent to participate was obtained from all of the adult participants and the parents or legal guardians of any participant under the age of 16.

Clinical and laboratory data were extracted anonymously, and genetic investigation was performed using whole-exome sequencing (WES). More specifically, genomic DNA was extracted from peripheral blood specimens and submitted to Macrogen Europe (Amsterdam, The Netherlands) for whole-exome sequencing. Exome capture was performed using the Agilent SureSelect Human All Exon V8 kit (Agilent Technologies, Santa Clara, CA, USA), and sequencing was conducted on the Illumina NovaSeq X platform (Illumina, Inc., San Diego, CA, USA). Approximately 6 Gb of sequencing data were generated per sample, achieving a mean target coverage exceeding 50×. Sequencing reads were aligned to the GRCh38 human reference genome, and standard bioinformatic pipelines were applied for variant calling of single-nucleotide variants (SNVs) and small insertions/deletions (indels). Functional annotation and generation of variant call format (VCF) files were performed by Macrogen Europe (Amsterdam, The Netherlands).

Bioinformatics analysis was subsequently conducted using the Franklin by Genoox platform. Variants were prioritized according to population frequency, predicted functional consequence, inheritance pattern, and phenotypic relevance. Rare variants (minor allele frequency < 0.01 in population databases) affecting genes associated with hereditary NDI and renal tubular disorders were specifically evaluated. Variant interpretation was performed according to American College of Medical Genetics and Genomics (ACMG) guidelines [16], integrating segregation data, clinical phenotype, previously published evidence, functional studies, computational predictions and information from public databases. No additional orthogonal validation was performed, as segregation analysis relied on high-confidence whole-exome sequencing data. The identified variant has been submitted to ClinVar and is publicly available under submission ID SUB16097905 and accession number SCV007539121 (University of Crete, Nephrology Department).

## 3. Results

### 3.1. Pedigree and Family Overview

**Figure 1:**
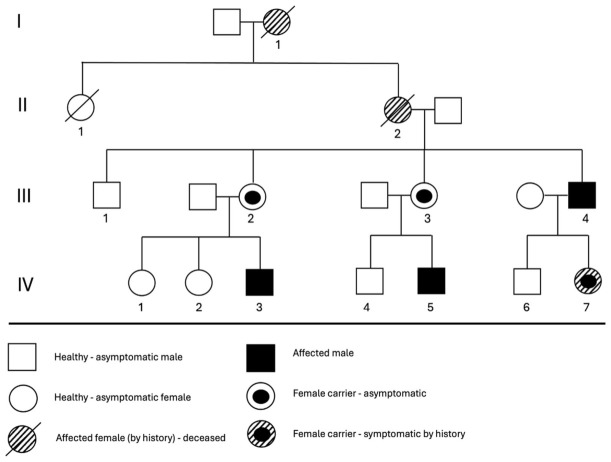
Family pedigree.

The study cohort comprised 14 individuals from 4 generations, including 11 genetically tested family members, along with 2 presumed obligate heterozygous females and 1 presumed healthy female without molecular data. The pedigree was consistent with X-linked inheritance (Figure 1). Molecular analysis identified the AVPR2 (NM_000054.7): c.964C>T variant segregating within the family, predicted to result in the p.Pro322Ser substitution. Genetic findings correlated with a heterogeneous clinical presentation, ranging from asymptomatic carriers to severely affected individuals with significant polyuria, electrolyte disturbances, and lower urinary tract complications (Table 1a,b). The maternal grandmother (I-1) reportedly had a history of kidney disease, although no molecular data were available. Her second daughter (II-2) had longstanding polyuria and is presumed to be an obligate heterozygote based on the presence of one affected son (III-4) and two carrier daughters (III-2 and III-3). In the subsequent generation, two males (IV-3 and IV-5) were affected, one female (IV-7) was a heterozygous symptomatic carrier, and the remaining individuals were unaffected.

**Table 1:** (**a**) Demographic and genetic characteristics of family members. (**b**) Clinical and laboratory characteristics of genetically affected and carrier individuals.

(a)														
Individual	Sex		Birth Year		Genetic Status		Inheritance Status			Age at Presentation (Years)		Clinical Phenotype		
I-1	F		1927		Not tested		Presumed affected			NA		Unclear renal disease		
II-1	F		1952		Not tested		Unaffected			None		Asymptomatic		
II-2	F		1955		Not tested		Presumed obligate heterozygote			15		Mild polyuria–polydipsia		
III-1	M		1971		Negative		Unaffected			None		Asymptomatic		
III-2	F		1972		Heterozygous		Carrier			None		Asymptomatic		
III-3	F		1975		Heterozygous		Carrier			None		Asymptomatic		
III-4	M		1980		Hemizygous		Affected			8		Partial NDI		
IV-1	F		1992		Negative		Unaffected			None		Asymptomatic		
IV-2	F		1996		Negative		Unaffected			None		Asymptomatic		
IV-3	M		1997		Hemizygous		Affected			5		Partial NDI		
IV-4	M		1999		Negative		Unaffected			None		Asymptomatic		
IV-5	M		2007		Hemizygous		Affected			<1		Complete (severe) NDI		
IV-6	M		2025		Negative		Unaffected			None		Asymptomatic		
IV-7	F		2021		Heterozygous		Symptomatic carrier			2		Mild polyuria–polydipsia		
(**b**)														
**Individual**	**Genetic Status**	**Clinical Phenotype**		**Age at Presentation (Years)**	**Daily Fluid Intake**	**Serum Sodium**		**Plasma Osm**	**Urine Osm After DDAVP Test**		**Hypokalemia**	**Renal Function**	**Urinary Tract Complications**	**Treatment**
III-2	Het	Asymptomatic		-	Normal	Normal		Normal	Not tested		No	Normal	None	None
III-3	Het	Asymptomatic		-	Normal	Normal		Normal	Not tested		No	Normal	None	None
III-4	Hem	Partial NDI		8	~11 L	Hypernatremia		Elevated	300–600 mOsm/kg		Yes	Normal	None	Thiazides, K suppl, low-salt diet
IV-3	Hem	Partial NDI		5	~12 L	Hypernatremia		Elevated	300–600 mOsm/kg		Yes	Normal	None	Thiazides, K suppl, low-salt diet
IV-5	Hem	Complete (severe) NDI		<1	~13 L	Hypernatremia		Elevated	<300 mOsm/kg		Recurrent	Normal	Pelvicalyceal dilatation, Mitrofanoff procedure, CIC × 5 daily	Thiazides, K suppl, low-salt diet
IV-7	Het	Mildly symptomatic		2	~3 L/m^2^	Normal		Normal	>800 mOsm/kg		No	Normal	None	None

Abbreviations: F, female; M, male; NDI, nephrogenic diabetes insipidus; NA, not available. Het, heterozygous; Hem, hemizygous; Osm, osmolality; DDAVP, 1-deamino-8-D-arginine vasopressin (desmopressin acetate); CIC, clean intermittent catheterization.; K suppl, potassium supplementation.

### 3.2. Individual Descriptions

I-1, a female born in 1927 and died in 1995, was reported to have a history of unclear renal disease with no molecular analysis performed.

II-1, a female born in 1952, was asymptomatic and died in 2025 with no kidney pathology. II-2, a female born in 1955 and died in 2023, has a longstanding history of mild polyuria and polydipsia since the age of 15 years, with a daily fluid intake of approximately 4 L, in the absence of electrolyte disturbances, renal impairment or treatment requirements. None of the females in generation II were genetically tested.

III-1, a male born in 1971, was asymptomatic and found to be negative for the identified variant following genetic testing prompted by the history of affected relatives. III-2 and III-3, females born in 1972 and 1975, respectively, were both asymptomatic and found to be heterozygous carriers of the variant. III-4, a male born in 1980, had a clinical diagnosis of NDI at the age of 8 years, with a daily water intake of approximately 11 L, hypernatremia with inappropriately diluted urine, hypokalemia and no other electrolyte disturbances, renal impairment or urinary tract complications. He was found to be hemizygous for the AVPR2 c.964C>T variant and remains stable on thiazide diuretics, potassium supplementation, and a low-salt diet.

IV-3, a male born in 1997, was diagnosed with NDI at the age of 5 years. He had a daily water intake of approximately 12 L, hypernatremia with inappropriately diluted urine, hypokalemia and no other electrolyte disturbances, renal impairment or urinary tract complications. Genetic analysis revealed hemizygosity for the detected variant and he remains stable on thiazide diuretics, potassium supplements and a low-salt diet. IV-5, a male born in 2007, presented with a severe NDI phenotype during the first year of life characterized by failure to thrive and recurrent episodes of dehydration. This was followed by excessive polyuria and polydipsia, recurrent hypokalemia and a daily fluid intake of approximately 13 L. He developed secondary pelvicalyceal dilatation and required a Mitrofanoff procedure, with bladder catheterization performed five times daily, while maintaining intermittent spontaneous voiding between catheterizations. Genetic testing confirmed hemizygosity for the familial AVPR2 c.964C>T variant and he remains stable with unimpaired renal function on thiazide diuretics, potassium supplements and a low-salt diet. IV-7, a female born in 2021, underwent genetic testing due to her affected father and was identified as a heterozygous carrier. Despite this, she was reported to have mild polyuria and polydipsia from the age of 2 years, with an estimated daily fluid intake of 3 L/m^2^, without other complications. IV-1, IV-2, IV-4, and IV-6, a female born in 1992, a female born in 1996, a male born in 1999, and a male born in 2025, respectively, were asymptomatic and found to be negative for the detected variant following genetic testing prompted by the history of affected relatives.

### 3.3. Genetic Findings and Variant Interpretation

Molecular analysis identified a familial AVPR2 (NM_000054.7):c.964C>T missense variant, inherited in an X-linked pattern and predicted to result in the p.Pro322Ser amino acid substitution. The variant co-segregated with disease among genetically tested individuals, whereas carrier status in two deceased females was inferred from pedigree analysis and clinical history. Variable phenotypic expression was observed among both hemizygous males and heterozygous females. The detected variant is not currently listed in the ClinVar database [12]; nevertheless, the p.Pro322Ser substitution has previously been suggested as causative in a Japanese family with a mild NDI phenotype [17].

According to ACMG/AMP guidelines [16], incorporating current ClinGen recommendations where applicable, the variant was classified as pathogenic. This classification was supported by moderate functional evidence demonstrating partial preservation of cAMP-mediated signaling for the p.Pro322Ser substitution (PS3) [18], absence from population databases (PM2) [19], the presence of other pathogenic missense variants affecting the same residue (PM5) [12], the strong co-segregation with disease among genetically confirmed family members (PP1), deleterious computational predictions (PP3) and a phenotype highly specific for AVPR2-related nephrogenic diabetes insipidus (PP4). In line with the above, the variant fulfills one strong and three moderate ACMG criteria, consistent with classification as pathogenic (Table 2).

## 4. Discussion

In this study, we describe a multigenerational Greek family with X-linked NDI associated with the AVPR2 (NM_000054.7):c.964C>T (p.Pro322Ser) variant. In the present family, the variant co-segregated with disease among genetically tested individuals—with additional support from obligate carrier status inferred through pedigree analysis—and was associated with a broad spectrum of clinical severity, ranging from asymptomatic heterozygous carriers to severely affected males with early-onset disease and urinary tract complications. These findings expand the clinical spectrum associated with this variant and provide further evidence supporting its pathogenic role.

Two of the three affected males (III-4 and IV-3) presented beyond infancy with normal development, absence of recurrent dehydration or urinary tract complications and evidence of residual urinary concentrating ability (urine osmolality after desmopressin acetate test 300–600 mOsm/kg), a picture consistent with partial NDI [9]. Our observations in two affected males are consistent with previous reports, in which p.Pro322Ser was associated with a milder clinical NDI phenotype [17] and, based on functional studies, partial preservation of cAMP-dependent signaling compared with other AVPR2 variants [18]. Interestingly, the third affected male (IV-5) had a severe early-onset disease, with recurrent episodes of hypernatremic dehydration and persistent inability to concentrate urine (urine osmolality after desmopressin acetate test < 300 mOsm/kg), complicated by urinary tract dilatation and required clean intermittent catheterization via a Mitrofanoff conduit while preserving spontaneous voiding between catheterizations. To our knowledge, such pronounced intrafamilial phenotypic variability has not previously been described among males carrying the p.Pro322Ser variant. In our cohort, one individual developed severe early-onset disease with urinary tract complications despite sharing the same molecular diagnosis as relatives with a substantially milder phenotype.

The remarkable intrafamilial variability observed in this family likely reflects a combination of biological and environmental factors beyond the primary AVPR2 defect. Differences in age at diagnosis, adherence to fluid intake recommendations, initiation of thiazide therapy and long-term management strategies may also affect clinical outcomes and the development of complications such as urinary tract dilatation [9]. Additional genetic modifiers influencing water handling, tubular transport, or vasopressin signaling pathways may contribute to disease severity [20]. Studies on chronic kidney disease have highlighted that disease expression and clinical outcomes often result from complex interactions among primary genetic defects, molecular pathways, environmental exposures, and individual susceptibility factors rather than from single variants alone [21]. Although derived from a different clinical setting, this conceptual framework may also be relevant to hereditary NDI and could help explain the marked intrafamilial variability observed in our family despite a shared AVPR2 genotype.

Heterozygous females demonstrated variable clinical expression, ranging from completely asymptomatic to mildly symptomatic individuals presenting with polyuria from early childhood or adolescence. This variability is most likely explained by skewed X-chromosome inactivation, which can lead to preferential expression of the mutant allele in renal collecting duct cells. Although X-linked NDI classically affects males, previous studies have reported symptomatic female carriers, some of whom exhibited a phenotype consistent with complete rather than partial NDI [22,23]. More recent studies have reinforced the role of skewed X-chromosome inactivation and other epigenetic mechanisms in determining disease severity among heterozygous females [13]. Symptomatic females in our family displayed relatively mild manifestations limited to polyuria and polydipsia without renal impairment or urinary tract complications. The evidence of symptomatic heterozygous females highlights the importance of careful clinical evaluation and long-term follow-up in female carriers, who are often considered unaffected.

The present study provides additional clinical and segregation evidence supporting the pathogenicity of the c.964C>T variant, although further functional studies and reports from independent families would strengthen this association. This interpretation is complemented by previously published functional studies indicating impaired receptor coupling due to p.Pro322Ser variant [18], reports of pathogenic substitutions affecting the same residue [17], and the highly specific phenotype observed in our family. The segregation observed in this family represents a particularly important contribution, as large multigenerational pedigrees are relatively uncommon in rare genetic disorders and provide robust evidence for variant pathogenicity. From a clinical perspective, our findings underscore the importance of early recognition and management of NDI, particularly in preventing complications such as dehydration, electrolyte disturbances and urinary tract dilatation, as emphasized in recent international recommendations for NDI diagnosis and management [9,24]. The severe phenotype observed in one individual highlights the potential long-term consequences of uncontrolled polyuria and emphasizes the need for close monitoring. Furthermore, identification of the causative variant enables targeted genetic counseling, early diagnosis in at-risk individuals and appropriate management strategies.

This study has several limitations. Functional characterization of the variant was not performed, and conclusions regarding its molecular effects are based on previously published data for similar variants. In addition, the study is limited to a single family, and further reports in independent cohorts would strengthen the evidence supporting pathogenicity.

## 5. Conclusions

In conclusion, our study describes a multigenerational Greek family in which the AVPR2 c.964C>T (p.Pro322Ser) variant was associated with substantial intrafamilial variability, including partial and severe manifestations among affected males and variable clinical expression among heterozygous females. Early genetic diagnosis enabled appropriate management, identification of at-risk relatives, and informed genetic counselling. The present findings contribute additional clinical and segregation evidence supporting the pathogenicity of this variant and underscore the importance of systematic family evaluation in hereditary nephrogenic diabetes insipidus.

## Figures and Tables

**Table 2:** Assertion criteria for pathogenicity by the type of evidence as per ACMG/AMP guidelines.

Evidence	Supporting	Moderate	Strong	Very Strong
Population Data		Absent in population database (*PM2*).		
Computational and Predictive Data	Computational prediction tools support a deleterious effect on the gene (*PP3*).			
Functional Data		Partial loss-of-function mechanism (*PS3*).		
Effect on Protein		Same residue as other pathogenic variants (*PM5*).		
Segregation Data			Co-segregation in multiple affected family members (*PP1*).	
Other Data	Highly specific phenotype for AVPR2-related NDI (*PP4*).			

## Data Availability

The identified variant has been submitted to ClinVar and is publicly available under submission ID SUB16097905 and accession number SCV007539121 (University of Crete, Nephrology Department).
